# DAXX and ATRX Expression in Canine Prostate and Bladder Cancer Identified by Immunohistochemistry—A Digital Quantitative Pilot Study

**DOI:** 10.3390/vetsci12121209

**Published:** 2025-12-17

**Authors:** Annika Spitzer, Heike Aupperle-Lellbach, Martin Spitzer, Silvia Weidle, Leonore Aeschlimann, Joshua Schwinn, Robert Klopfleisch, Simone de Brot

**Affiliations:** 1 LABOKLIN GmbH&Co. KG, 97688 Bad Kissingen, Germany; a.spitzer@laboklin.com (A.S.); spitzer@laboklin.com (M.S.); schwinn@laboklin.com (J.S.); 2 Institute of Pathology, School of Medicine, Technical University of Munich, Trogerstr. 18, 80333 München, Germany; silvia.weidle@tum.de; 3 Institute of Animal Pathology, Comparative Pathology Plattform (COMPATH), University of Bern, 3012 Bern, Switzerland; leonore.kuechler@unibe.ch (L.A.); simone.debrot@unibe.ch (S.d.B.); 4 Institute of Veterinary Pathology, Free University of Berlin, 14195 Berlin, Germany; robert.klopfleisch@fu-berlin.de

**Keywords:** dog, molecular profiling, DAXX, ATRX, immunohistochemistry, digital

## Abstract

The chromatin remodelling proteins DAXX and ATRX are key regulators of genome stability and epigenetic processes. Alterations in their expression have been associated with tumour stage and prognostic outcomes in various human cancer types, whereas their role in veterinary oncology has received little investigation to date. We analysed canine prostate and urinary bladder samples, including 18 prostate carcinomas (12 adenocarcinomas and 6 prostatic urothelial carcinomas), 10 non-malignant prostate tissues, 22 urinary bladder carcinomas, and 6 non-malignant bladder tissues. Nuclear expression of DAXX and ATRX was assessed using fully quantitative digital immunohistochemistry. Overall, DAXX exhibited consistently higher expression than ATRX across benign and malignant samples from the prostate and bladder. DAXX and ATRX expression demonstrated a positive correlation across all samples (ρ = 0.553, *p* < 0.05), suggesting coordinated regulation. They displayed organ-specific expression patterns: prostate carcinomas showed increased expression of DAXX compared with non-malignant prostate tissues (*p* < 0.05). In contrast, in the bladder, expression of DAXX and ATRX declined with increasing tumour grade (*p* < 0.05). Our findings provide new insights into the potential of DAXX and ATRX as biomarkers, offering new insights into their clinical relevance in dogs.

## Simple Summary

DAXX and ATRX are nuclear proteins that play a central role in preserving genome stability and regulating gene expression through chromatin organisation. In human oncology, alterations in the expression of these proteins have been associated with tumour aggressiveness and a poor prognosis for various cancer types. In this study, we examined benign and malignant tissues from the canine prostate (*n* = 28) and urinary bladder (*n* = 28) to investigate the expression of DAXX and ATRX. Digital quantitative immunohistochemistry revealed a positive correlation between DAXX and ATRX expression across all samples. Moreover, prostate carcinomas showed increased expression of DAXX compared with non-malignant tissues. In contrast, urothelial carcinomas of the bladder displayed reduced expression of DAXX and ATRX in tumours with increased aggressiveness. Our findings provide a better understanding of how DAXX and ATRX expression is altered in canine prostate and bladder tumours, indicating that their potential clinical relevance should be further investigated.

## 1. Introduction

As neoplastic diseases represent the leading cause of death in dogs [1], a thorough understanding of the underlying pathomechanisms is essential to make informed clinical decisions. In oncology, tumour biomarkers hold significant value for diagnosis, prognosis, and the development of targeted therapies [2]. Biomarkers are defined as characteristics of neoplastic processes that can be objectively measured and evaluated as indicators of pathogenic biological activity [3]. Among the numerous molecular biomarkers under investigation, the chromatin remodelling proteins DAXX (death-domain associated protein) and ATRX (alpha-thalassemia/intellectual disability syndrome X-linked) have gained increasing attention [4,5]. They play a role in epigenetic regulation, genomic stability, and telomere maintenance [6,7]. Among their various functions [8,9], DAXX and ATRX form a complex which is responsible for the deposition of the histone variant H3.3 at repetitive regions of the genome, especially telomeric and pericentromeric DNA [6]. In healthy cells, the proper attachment of the ATRX/DAXX/Histone3.3 complex to these regions is essential for genome stability and heterochromatic architecture, as it prevents aberrant transcription through restricting transcriptional processes [10].

Alterations in the expression of DAXX and/or ATRX can influence tumour biology. Furthermore, they are correlated with tumour stage and prognostic outcomes across different human cancer types, including meningiomas, melanomas, various sarcomas, and neuroendocrine neoplasms [8,11]. Among these, pancreatic neuroendocrine tumours (PanNENs) are the best investigated neoplasms in this respect to date [12]. The loss of DAXX and ATRX expression is associated with shorter patient survival and more aggressive tumour behaviour in PanNENs [13]. Thus, immunohistochemistry for these markers is included in routine molecular diagnostic panels to support risk stratification and clinical decision-making in human medicine [14]. In contrast, Vries et al. already showed that canine PanNENs differ from their human counterparts regarding their ATRX gene expression, although the majority of canine PanNENs (~90%) are aggressive carcinomas in dogs [15]. No loss of ATRX expression was observed in canine PanNENs [16], in contrast to the frequent loss (in ~36% of cases) reported in human PanNENs [17].

Furthermore, in veterinary medicine, Kreilmeier et al. investigated DAXX and ATRX expression in the context of alternative lengthening of telomeres (ALT) in various canine sarcomas [18]. Loss of ATRX expression was detected in haemangiosarcoma (1 of 8), histiocytic sarcoma (2 of 5), osteosarcoma (1 of 4), multilobular osteochondrosarcoma (1 of 1), sticker sarcoma (1 of 2), fibrosarcoma (1 of 15), and one undifferentiated sarcoma (1 of 3). In contrast, loss of DAXX expression was not observed. In addition, Wong et al. reported ATRX mutations in 4 of 15 canine visceral haemangiosarcomas [19]. Mutations of DAXX and ATRX appeared to correlate with loss of nuclear immunolabelling, as seen in PanNENs [13,20], although exceptions may occur depending on the tumour type [21].

To date, no veterinary studies immunohistochemically investigated DAXX or ATRX expression in canine neoplasms other than PanNENs [16] and sarcomas [18]. Nevertheless, their potential value as molecular tumour markers may also be relevant in canine prostatic and urothelial carcinomas. In men, alterations in DAXX and/or ATRX expression were reported for both prostate and urinary bladder carcinomas. In human prostate carcinomas, strong DAXX protein expression has been shown to correlate with higher malignancy (e.g., higher Gleason grades) and increased tumour cell proliferation [22]. Immunohistochemical studies assessing nuclear ATRX expression in the prostate remain to be investigated. However, ATRX mRNA was found to be significantly downregulated in human prostate carcinomas [23]. In human urinary bladder carcinomas, DAXX expression was characterised by reduced nuclear staining intensity compared with normal urothelium [24]. The loss of expression was associated with higher tumour stage [24]. Studies specifically assessing ATRX protein expression in urinary bladder carcinomas are currently lacking. To date, DAXX and ATRX immunohistochemistry have not been incorporated into routine molecular diagnostic panels for prostate or urinary bladder carcinomas in human oncology.

In pathological practice, formalin-fixed paraffin-embedded (FFPE) tissue samples are the most commonly available material for diagnostic and research purposes. Therefore, immunohistochemistry (IHC) is an ideal approach for estimating protein expression and identifying potential new biomarkers, such as DAXX and ATRX. Both antibodies used in this study were established for canine tissues and previously examined in canine sarcomas [18] and pancreatic neuroendocrine neoplasms [16]. As DAXX and ATRX primarily exert their biological functions within the nucleus, nuclear expression is considered most relevant for immunohistochemical evaluation. ATRX expression is consistently described as nuclear. In contrast, cytoplasmic localisation of DAXX has been observed in certain human tumour types, for example, in gastric cancer [25], and may carry additional biological significance [26].

The evaluation of IHC-stained tissue slides traditionally relies on visual assessment of staining intensity. However, this approach can be subject to a certain degree of variability. Even among experienced pathologists, both interobserver [27,28] and intraobserver variability [29] were reported. To address these limitations, digital pathology is becoming increasingly important for the evaluation and quantification of tissue biomarkers. Automated analysis of scanned tissue slides, frequently aided by artificial intelligence (AI) algorithms, can help pathologists produce results that are more objective, quantitative, and reproducible [30]. The evaluation of IHC-based tumour markers—both quantitatively and qualitatively—has become a prominent and effective application of digital and AI-driven pathology [31]. While digital IHC analysis of whole-slide images is increasingly used, standardised analytical protocols exist for only a small group of the most extensively studied tissue markers, including Ki-67 [32], HER2 [33] and oestrogen and progesterone receptor for breast cancer [34]. Very few digital analytic programmes have yet been approved by the U.S. Food and Drug Administration (FDA) or the European In Vitro Diagnostic Regulation (IVDR), underscoring that clinical implementation of automated IHC quantification is still in development. Current digital analysis programmes are offered by vendors such as Visiopharm, Indica Labs, Leica Biosystems, Roche Diagnostics, and Philips Healthcare.

This study aims to investigate alterations in the epithelial nuclear expression levels of DAXX and ATRX in canine carcinomas of the prostate and bladder using immunohistochemical methods combined with AI-assisted digital image analysis.

## 2. Materials and Methods

### 2.1. Samples

Formalin-fixed, paraffin-embedded (FFPE) samples of benign and malignant prostate and urinary bladder tissues were available from 56 dogs. Included were 18 prostate carcinomas (PC; 12 adenocarcinomas, six prostatic urothelial carcinomas), 10 non-malignant prostate samples, 22 urothelial carcinomas of the bladder (UC), and 6 non-malignant bladder samples. An overview of included cases is provided in Supplementary Table S1.

Samples originated from Laboklin GmbH and Co. KG (Bad Kissingen, Germany) and the Institute of Animal Pathology at the University of Bern (Bern, Switzerland), and were retrieved from archival diagnostic material collected between 2003 and 2022. Submission of samples included consent for research use, as specified on the respective submission forms.

### 2.2. Histology and Tissue Microarray (TMA)

Haematoxylin and eosin (HE)-stained slides were available from each sample. General inclusion criteria required an unambiguous histological diagnosis, good quality of the sample (e.g., no crimping), and enough material. Based on HE-stained whole tissue sections, all examined UC originated from the transitional epithelium of the urinary bladder. In contrast, PC were classified as either adenocarcinomas or prostatic urothelial carcinomas, depending on histology and the reported sampling site, following the system proposed by Palmieri et al. [35]. Furthermore, PC were histologically graded using the modified Gleason score [36], ranging from 2 to 10, with score 10 representing the most aggressive and poorly differentiated PC. The Gleason score was applied only to cases in which architectural Gleason patterns could be reliably identified; cases of prostatic urothelial carcinomas remained ungraded. UCs of the urinary bladder were graded as Grade 1, 2, or 3 based on the system described by Valli et al. [37], with Grade 1 representing well-differentiated and Grade 3 highly anaplastic UC. For information on the histological grading of each case, please refer to Supplementary Table S1.

Two tissue microarray (TMA) blocks were created, one for prostate samples and one for urinary bladder samples, with core diameters of 2 mm and containing 1 to 6 cores from each case. TMA blocks were manufactured using the TMA Grand Master (3DHistech, Budapest, Hungary) at the Comparative Experimental Pathology department of the Institute of Pathology at the Technical University of Munich in Germany. For each sample, regions of interest were identified on the HE-stained whole tissue sections and subsequently manually transferred to the donor blocks. Where applicable, tissue cores were selected to capture different tumour growth patterns and adjacent non-neoplastic areas. If the tumour tissue showed only one histomorphological growth pattern (e.g., solid), only one representative core was selected. If different growth patterns were visible (e.g., tubular and solid), cores from both patterns were transferred to the recipient block. Furthermore, if normal tissue was available, it was sampled additionally.

### 2.3. Immunohistochemistry (IHC)

TMA sections were HE-stained before immunohistochemical analyses. DAXX and ATRX immunohistochemistry were performed at the Technical University of Munich on SuperFrost slides (Langenbrinck, Emmendingen, Germany) from the TMA blocks. Both antibodies were anti-rabbit polyclonal IgG antibodies from Sigma Aldrich^®^ (Darmstadt, Germany) (DAXX: HPA008736; ATRX: HPA001906). For antigen retrieval, slides were prepared using Tris-EDTA buffer for 30 min for DAXX and citrate buffer for 20 min for ATRX. Staining for both antibodies was performed using a Bond RXm system (Leica Microsystems, Wetzlar, Germany) with the Bond Polymer Refine Detection Kit (Leica Microsystems, Wetzlar, Germany). The staining protocol began with a 5 min peroxide block (Refine Detection Kit Peroxide Block) to suppress endogenous peroxidase activity, followed by a 15 min incubation with the primary antibody. Next, an 8 min incubation with a polymer-based secondary detection system (Refine Detection Kit Polymer) was performed, followed by chromogen development with Diaminobenzidine (Refine Detection Kit Mixed DAB Refine) for 10 min. Finally, slides were counterstained with haematoxylin for 5 min (Refine Detection Kit Haematoxylin).

Human tonsil tissue was used as a positive control for DAXX, and human glioblastoma tissue as a positive control for ATRX. Prostate and bladder TMA slides were stained in the same run for both antibodies. Simultaneously with the immunohistochemical staining, slides from both TMAs were prepared without the primary antibody to serve as a negative reagent control (NRC).

### 2.4. Digital IHC Quantification

Following HE and IHC staining, the glass slides were scanned at 40× resolution using the S360 Nanozoomer (Hamamatsu Photonics, Shizuoka, Japan). Digital assessment of DAXX and ATRX IHC staining was performed by two board-certified veterinary pathologists (S.d.B., L.A.) and a pathology resident (A.S.) using Visiopharm software (version 2025.02 x64, Hørsholm, Denmark). The following workflow was applied: (1) Tissue detection and segmentation into epithelial and stromal (deep learning (DL) classification, U-Net; input magnification 10×; >600k iterations). This classifier was reused from a previous study and was trained on annotations from 35 canine prostate and bladder tissue sections stained for a nuclear antigen (oestrogen receptor alpha IHC). Epithelium (benign or malignant) was defined as regions of interest (ROI); (2) Revision of ROIs with manual corrections where needed; (3) Detection and labelling of individual epithelial cell nuclei (predefined Visiopharm #10173 IHC Nuclei Detection APP; DL classification, U-Net; input magnification 20×; >100k iterations); (4) Generation of output: total nuclear HDAB-DAB pixel values range 0 (dark brown; strong immunolabeling) to 255 (white; no immunolabeling) per TMA core (mean, median, standard deviation). After analysis, each TMA core was reviewed for the quality of epithelial nuclear segmentation, the presence of non-specific staining, and potential tissue artefacts. Nuclear segmentation was consistently precise across all samples, defined as fewer than 1% of nuclei being undetected or fused. Non-specific staining was limited to weak to moderate DAXX immunoreactivity in stromal leucocytes. Importantly, leucocyte infiltration into the epithelium was absent or negligible. This stromal background staining did not affect the DAXX or ATRX assessment, as only epithelial regions were analysed and stromal areas were excluded. Rare instances of tissue artefacts not captured during the previous tissue segmentation were manually corrected by excluding these regions from the ROI after which the slide was reassessed.

### 2.5. Statistical Analysis

For all calculations, the mean HDAB-DAB pixel value of all epithelial nuclei within each TMA core as provided by the Visiopharm software was used as the raw data. As for some of the cases multiple TMA cores were analysed, aggregated mean values were then calculated for each case as the unweighted average of the means from all TMA cores belonging to that specific case. Consequently, all cores were considered equally representative for the respective case. This resulted in 28 records for the prostate samples as well as 28 for the bladder samples with a mean pixel value for ATRX expression and DAXX expression each. However, three prostate samples were flagged as unreliable by the Visiopharm software, leaving 25 prostate records suitable for statistical analysis. Furthermore, the ATRX value for case 4 and the DAXX value for case 56 were also flagged as unreliable and were therefore excluded from the analysis (see Supplementary Table S1).

For visualisation purposes, the aggregated mean values were linearly inverted (x′ = 255 − x) so that higher values corresponded to stronger immunohistochemical staining intensity. This monotonic transformation preserved relative differences and statistical relationships while enhancing interpretability. The inverted mean values per case are summarised in Supplementary Table S1.

Prostate samples were grouped into non-malignant tissues, prostatic urothelial carcinomas (PUCs) and prostatic adenocarcinomas (PACs). Due to the low number of PUC samples, PUCs and PACs were summarised as prostate carcinomas, leaving two groups for further statistical analysis of prostate samples. Bladder samples were grouped into following groups: (1) non-malignant bladder tissues; (2) urothelial carcinomas (UC) Grade 2; (3) UC Grade 3.

All statistics were generated in R (Version 4.5.1). Overall, the data did not show a normal distribution, as assessed by the Shapiro–Wilk test. Spearman’s rank correlation was used to evaluate the relationship between DAXX and ATRX expression across all samples, independent of their group.

Inverted HDAB-DAB values (i.e., brown IHC expression levels) were compared separately for DAXX and ATRX. Groups from the bladder (non-malignant bladder samples, UC Grade 2, UC Grade 3) were compared using the Kruskal–Wallis test. For pairwise comparison of bladder groups Dunn’s test was used as post hoc test. Groups of the prostate (non-malignant prostatic samples, prostate carcinomas) were compared using the Mann–Whitney U test. Statistical significance was set at *p* < 0.05. To control for statistical type 1 error, Bonferroni correction was conducted by multiplying *p*-values by the number of tests applied within each variable (DAXX and ATRX; k = 5).

## 3. Results

### 3.1. Histomorphological Evaluation in HE-Stained Sections

Among the prostate samples (*n* = 28), eighteen were classified as prostate carcinoma (PC). Of all PC, twelve were identified as adenocarcinomas originating from the glandular epithelium of the prostate. Six samples were classified as prostatic urothelial carcinomas arising from the prostatic urethra or the prostatic ducts near the urethra. All adenocarcinomas displayed mixed solid and tubular growth patterns, except for one case (case 9), which had a predominantly tubular pattern. Intravascular tumour cells were detected in three adenocarcinomas (cases 1, 2, and 5). Using the veterinary Gleason Scoring system [36], all adenocarcinomas exhibited aggressive histological patterns, ranging from fused glands and vacuolated cytoplasm to solid sheets, cords, or single cells without glandular differentiation, as well as comedonecrosis. Consequently, four adenocarcinomas were assigned a Gleason score of 9 (cases 1–4), and eight samples were assigned a Gleason score of 10 (cases 5–12). The number of Gleason score 9 cases in our cohort was relatively small (*n* = 4). Therefore, differences between adenocarcinomas with Gleason scores 9 and 10 were not further evaluated in this study. Similarly, in human medicine, Gleason scores 9 and 10 are both categorised as Grade Group 5 and are considered to have comparable prognostic behaviour [38].

All six prostatic urethral carcinomas exhibited typical urothelial morphology, without any further subdivision possible. Non-malignant prostate samples included two cases of benign prostatic hyperplasia (cases 26 and 27), one atrophic prostate (case 28), four prostates from juvenile dogs (cases 22–25), and three samples of normal prostate tissue (cases 19–21).

Among the urinary bladder samples (*n* = 28), twenty-two were classified as urothelial carcinomas of the bladder (UC), exhibiting clear signs of malignancy, including disorganised growth, variation in cell and nuclear size, often with irregular nuclear crowding and moulding, as well as prominent nucleoli. In the most aggressive cases (cases 46–50), muscular invasion and numerous, partly abnormal, mitotic figures could be observed.

The predominant growth pattern was papillary in 13 cases, solid in eight cases, and mixed solid and papillary in one case (case 38). Intravascular tumour cells were detected in two papillary UC (cases 32 and 47) and in the single mixed solid-papillary UC (case 38). According to the grading system from Valli et al. [37], seventeen UC were assigned Grade 2 (cases 29–45), and five were UC Grade 3 (cases 46–50), while no cases met the criteria for Grade 1. Non-malignant bladder samples included three papillomas (cases 54–56), one hyperplasia (case 53), and two samples of histologically normal urothelium (cases 51 and 52).

Detailed information for each case, including the dog’s breed, sex, and age, is provided in Supplementary Table S1.

### 3.2. Overall DAXX and ATRX Expression in Prostate and Bladder Tissues

The DAXX and ATRX antibodies showed valid and interpretable immunolabeling. Expression of both proteins was indicated by a variably intense brown colour, predominantly localised to the nuclei of positive cells. In general, DAXX showed stronger expression compared to ATRX, with median HDAB-DAB pixel values of 173 and 53, respectively. In cases with very strong expression, the brown colour extended into the cytoplasm, which was frequently seen in cases with a strong DAXX expression. Even in such cases, however, nuclear expression remained clearly distinguishable.

DAXX and ATRX expression levels showed a moderate, significant positive correlation across all samples as assessed by Spearman’s rank correlation (ρ = 0.553, *p* < 0.001). However, for both markers and both anatomical sites, mild intratumoral heterogeneous expression intensity was frequently observed. Three samples (case 3, 17, and 18) were negative for both markers. However, there was no association between the age of the samples and the reactivity in immunohistochemistry.

For DAXX, expression intensity in the interstitial connective tissue varied depending on the presence and extent of inflammation. Stromal cells showed little to no expression, whereas leucocytes were DAXX-positive with variable expression intensity. For ATRX, the expression in stromal cells and leucocytes was generally negative or only weakly positive. Since epithelial nuclear expression was considered more relevant for assessing alterations of DAXX and ATRX in carcinomas, variations in stromal expression and inflammatory cells were not further analysed.

### 3.3. Epithelial Nuclear DAXX and ATRX Expression in the Prostate

Inverted HDAB-DAB pixel values of DAXX were significantly higher in prostate carcinomas compared with prostate non-malignant tissue (*p* = 0.010). For ATRX no significant differences in inverted HDAB-DAB pixel values were observed across the two groups (*p* > 0.05). The median of inverted HDAB-DAB pixel values of the non-malignant group was 138 for DAXX and 23 for ATRX with a standard deviation (SD) of 22.6 for DAXX and 14.7 for ATRX. For the PC group the median was 177 for DAXX and 39 for ATRX with a SD of 18.5 for DAXX and 19.8 for ATRX.

**Figure 1:**
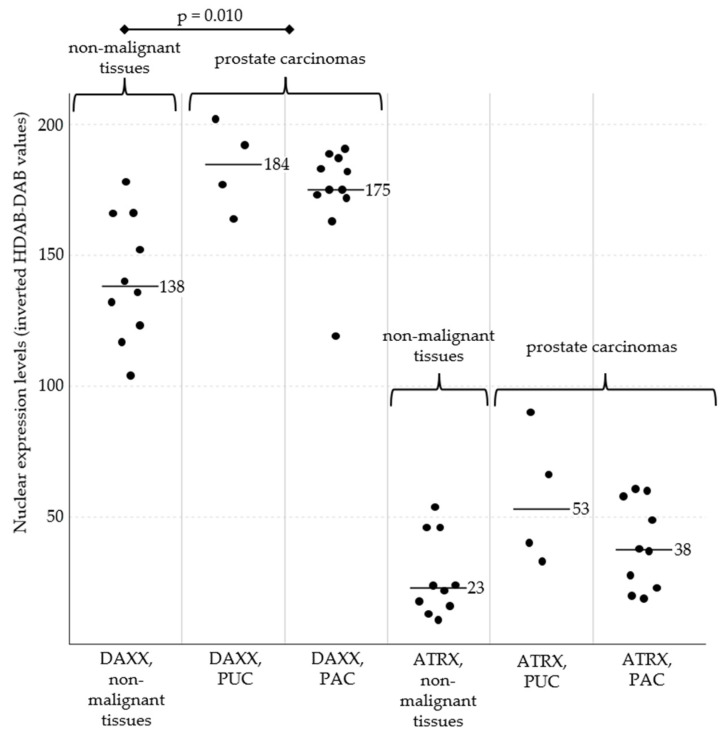
Nuclear expression of DAXX (**left**) and ATRX (**right**) in prostate tissues. The inverted HDAB-DAB pixel value of DAXX was significantly higher in the neoplastic prostate gland compared to the non-malignant group (*p* = 0.010). For ATRX, no significant differences were observed (*p* > 0.05). Across all samples, DAXX expression was generally stronger than ATRX expression. Between PUC and PAC, no significant differences for DAXX and ATRX in pixel values were observed (*p* > 0.05). Black bars represent the median expression values for each subgroup. Abbreviations: PUC, prostatic urothelial carcinomas; PAC, prostatic adenocarcinomas.

**Figure 2:**
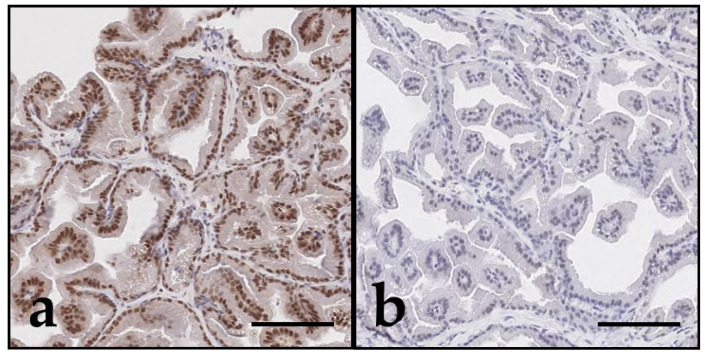

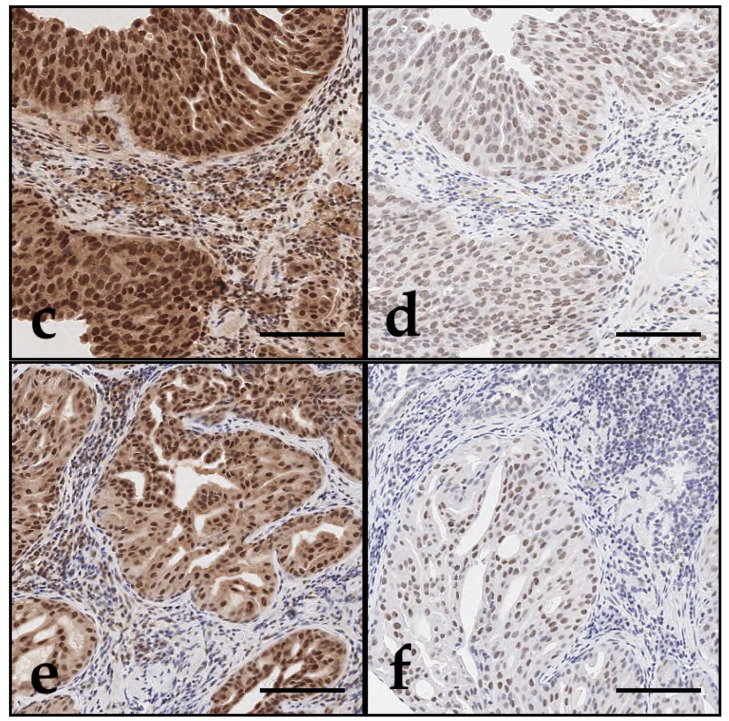
DAXX and ATRX immunohistochemistry of canine prostate tissue; bar indicates 100 µm. Immunostaining with both antibodies showed predominant nuclear expression of DAXX and ATRX in epithelial cells, visualised by brown nuclear staining. DAXX (**a**,**c**,**e**) showed overall stronger expression compared to ATRX (**b**,**d**,**f**) across all prostate samples. PC (**c**–**f**) showed stronger DAXX and ATRX expression compared to benign prostatic conditions such as hyperplasia ((**a**,**b**); case 20). This increase was observed in both prostatic urothelial carcinoma ((**c**,**d**); case 15) and adenocarcinoma ((**e**,**f**); case 7), and was particularly pronounced for DAXX. Intratumoral heterogeneity in the expression of epithelial DAXX and ATRX was observed, represented by variable expression intensity of individual tumour cell nuclei.

Expression intensity was notably stronger in neoplastic tissue than in the non-malignant group (Figure 1 and Figure 2). This was particularly evident in the case of the DAXX expression. However, one individual case of prostatic adenocarcinoma (case 8) deviated from this pattern, showing histological evidence of malignancy despite relatively weak expression of both markers.

No differences in pixel values could be determined between different dog breeds or histological types (adenocarcinoma (PAC) vs. prostatic urothelial carcinoma (PUC)).

### 3.4. Epithelial Nuclear DAXX and ATRX Expression in the Bladder

**Figure 3:**
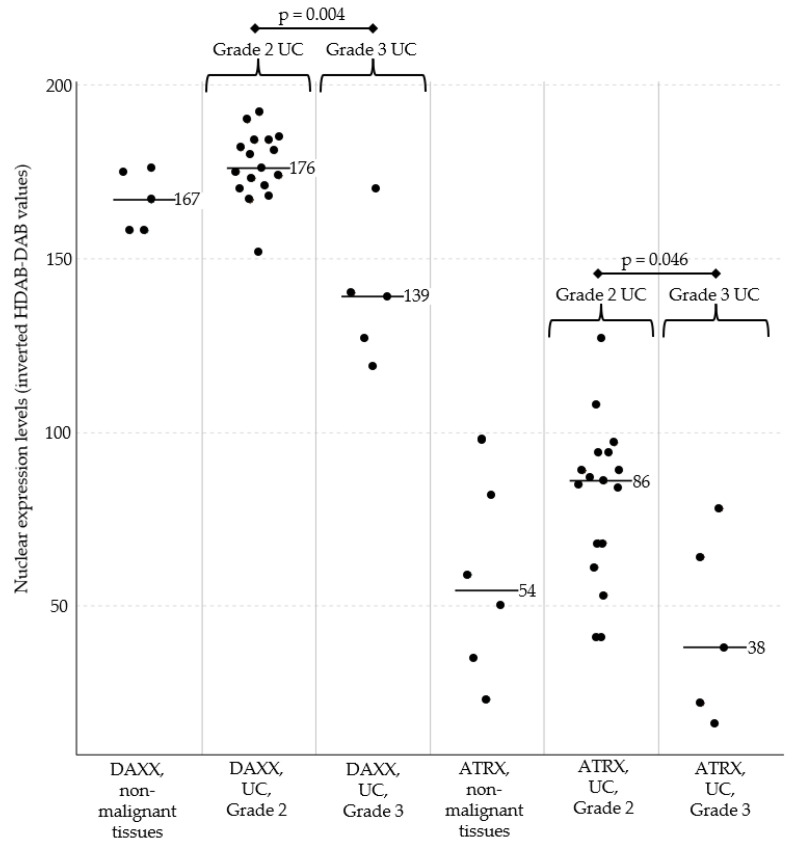
Nuclear expression levels of DAXX (**left**) and ATRX (**right**) in urinary bladder tissues. DAXX and ATRX expression did not differ significantly between all urothelial carcinomas (UC) and non-malignant bladder tissues. The more aggressive Grade 3 UC showed significantly lower expression of DAXX (*p* = 0.004) and ATRX (*p* = 0.046) compared to Grade 2 UC. Across all samples, DAXX expression was generally stronger than ATRX expression. Black bars represent the median expression values for each subgroup. Abbreviations: UC, urothelial carcinomas of the urinary bladder.

Compared to control urothelium, no significant differences in DAXX and ATRX inverted pixel values were observed in urothelial carcinomas (*n =* 22). However, the inverted HDAB-DAB pixel value was significantly lower in grade 3 UC compared to grade 2 UC for both DAXX (*p* = 0.004) and ATRX (*p* = 0.046) (Figure 3).

The median of inverted HDAB-DAB pixel values of non-malignant tissue was 167 for DAXX and 54 for ATRX with a SD of 7.8 for DAXX and 22.5 for ATRX. The median of UC Grade 2 was 176 for DAXX and 86 for ATRX with a SD of 19.7 for DAXX and 27.4 for ATRX. The median of UC Grade 3 was 139 for DAXX and 38 for ATRX with a SD of 17.3 for DAXX and 23.9 for ATRX. ATRX showed overall a higher SD.

On histological examination of the HE-stained slides, UC with reduced expression were characterised by high cellular and nuclear pleomorphism and an invasive growth pattern. In contrast, well-differentiated UC with preserved organoid architecture (corresponding to Grade 2) exhibited noticeably stronger expression.

**Figure 4:**
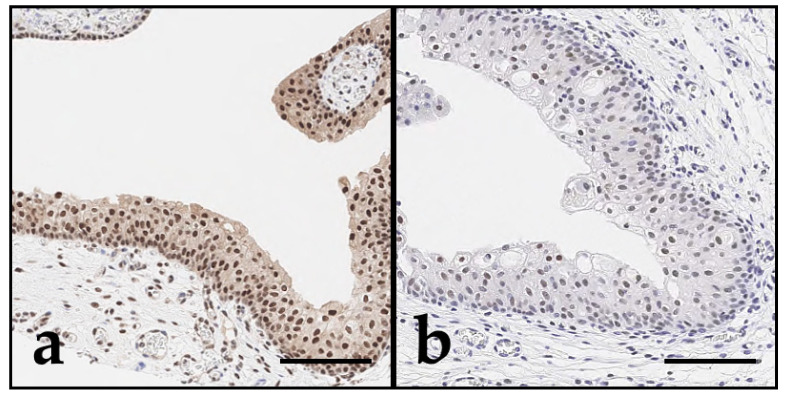

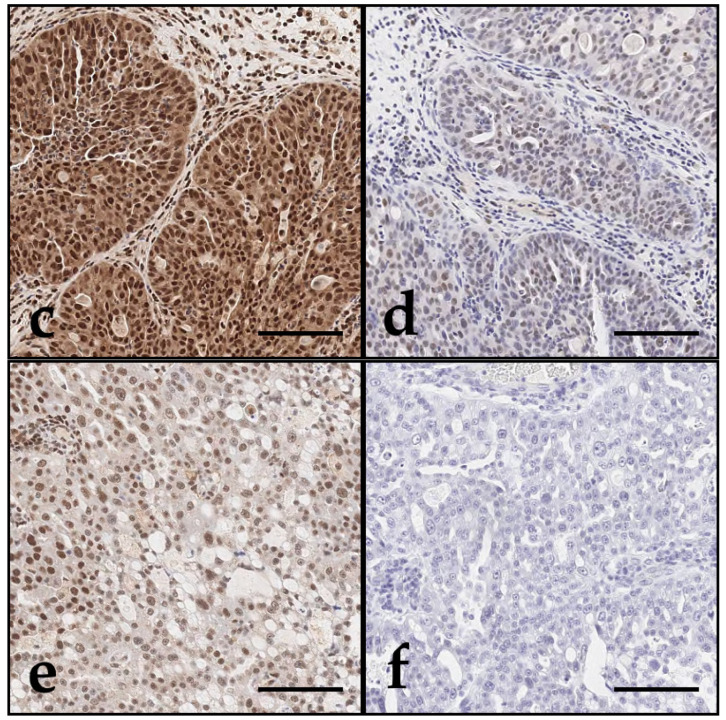
DAXX and ATRX immunohistochemistry of canine bladder tissue; bar indicates 100 µm. Across all bladder samples, DAXX (**a**,**c**,**e**) showed overall stronger expression compared to ATRX (**b**,**d**,**f**), as visualised by variable brown nuclear staining. While Grade 2 UC ((**c**,**d**); case 45) tended to show stronger expression compared with non-malignant urothelium ((**a**,**b**); case 52), expression of both DAXX and ATRX decreased in the more aggressive Grade 3 UC ((**e**,**f**); case 49). Within tumours, expression heterogeneity was observed, with loss of nuclear immunolabelling for DAXX and ATRX in regions with increased tumour cell pleomorphism (**e**,**f**).

In summary, Grade 2 UC exhibited higher expression intensity of DAXX and ATRX, whereas these markers were reduced in the more invasive and pleomorphic Grade 3 UCs (Figure 4). No significant difference in expression intensity was observed for DAXX or ATRX among dog breeds.

## 4. Discussion

This pilot study aimed to investigate the expression of DAXX and ATRX in urothelial and prostate carcinomas, as well as in non-neoplastic tissues. In summary, the patterns of DAXX and ATRX expression varied between non-malignant samples and tumour tissues in the prostate. Prostate carcinomas exhibited a significant increase in the expression of DAXX compared to non-malignant samples. In the urinary bladder, Grade 2 UC tended to show stronger expression than non-malignant tissues, although this did not reach statistical significance. Interestingly, the more aggressive Grade 3 UC displayed an apparent reduction in expression of both markers compared with the less malignant Grade 2 UC.

### 4.1. Methodological Considerations

Several methodological aspects of this study must be considered when interpreting the results. The use of AI-based digital IHC quantification enabled the objective evaluation of nuclear expression levels, minimising interobserver or intraobserver variability. This was especially helpful for evaluating ATRX expression, where the generally weaker staining intensity could have made it difficult to recognise subtle differences by eye alone. The method also enabled precise and reproducible quantification of expression intensity, capturing variations that exceed the limits of visual assessment.

Nevertheless, the use of AI in immunohistochemical evaluations is not without limitations. Pre-analytic factors—including variability in slide preparation, staining quality, and scanner settings—can affect algorithmic performance, which is why quality control by an experienced pathologist remains essential to ensure reliable results [39,40]. On the analytic side, automated digital IHC assessment still faces several persistent challenges that hinder standardisation and limit cross-study comparability. These include inconsistencies in region-of-interest selection, difficulties in accurately segmenting tumour tissue and individual cells, subjective thresholds for defining marker positivity, methodological differences between manual and automated counting approaches, and the lack of consensus regarding the most informative quantitative outputs [41,42,43]. Together, these pre-analytic and analytic sources of variability underscore the need for harmonised workflows and robust validation strategies. In the context of the present study—and indeed for any investigation relying on fully or semi-automatic quantitative tissue-marker assessment—it is essential to clearly define all of the above pre-analytic and analytic parameters and to apply them consistently across all samples. Only by performing the analysis with identical settings for every slide can variability be minimised and the resulting measurements rendered comparable and reproducible. In this study, all assessed tissue cores were placed on a single slide, ensuring that staining and scanning were performed under fully identical conditions. As the region of interest, we defined the entire available epithelium, thereby avoiding selective hotspot analysis. Staining intensity was evaluated on a continuous scale directly on the native digital image, without imposing thresholds or categorising cells into arbitrary positivity classes.

The use of TMAs is advantageous, as it enables uniform processing conditions across large sample cohorts, thereby reducing technical variability. The DAXX and ATRX antigens appeared to be stable, and no correlation was observed between the age of the samples and immunohistochemical reactivity. The methods of paraffin embedding have remained unchanged in our laboratories throughout the entire study period. However, the sample handling (concentration and duration of formalin fixation) by clinicians cannot be fully standardised, which may explain the three non-reactive samples. This is a challenge that must be addressed in any study aiming to develop tests under the routine conditions of veterinary pathology diagnostics [44].

The selection of cores with small diameters may lead to overlooking the phenomenon of intratumoral heterogeneity. However, we observed that some variable expression intensity exists between different growth patterns within some samples, indicating mild intratumoral expression heterogeneity in these markers. Furthermore, we decided to focus on the evaluation of the epithelial component of the tissue because in the cores, it was not possible to map the interstitial and inflammatory components representatively. Other studies focused on the immune landscape of the canine bladder [45]. Thus, further studies on full-size samples, utilising spatial techniques, should address this to evaluate the environmental effects on oncogenesis and protein expression in more detail.

Interestingly, we had no grade 1 urothelial carcinomas in our cohort that met the inclusion criteria. The number of grade 1 urothelial carcinomas is generally low in dogs [37,46]. However, improved diagnostic methods, including BRAF analysis in urine [47,48] may result in more cases of the early stage of malignant transformation [49]. Thus, if available, future studies should include grade 1 urothelial bladder carcinomas to cover this aspect. Furthermore, rare histological subtypes as described by Lin et al. [50] should be included in further studies.

Both antibodies used in this study demonstrated reliable immunolabeling, confirming their suitability for use in canine tissues and underlining the results from previous studies [16,18]. The heterogeneity of the tissue collection in our research can be viewed as both a strength and a limitation. This pilot study allowed us to observe DAXX and ATRX expression encompassing different groups of samples (benign vs. malignant; various growth patterns). However, the representativeness of the immunohistochemical results was limited because of the small number of cases per group. Nevertheless, the consistently correlated expression patterns of DAXX and ATRX strongly suggest that our findings reflect an actual biological effect rather than an artefact of sample variability.

The fact that this study primarily focused on methodological aspects and used retrospective material without additional clinical data limited the interpretation. However, future studies may address topics such as prognostic or therapeutic aspects in more detail.

### 4.2. Organ-Specific Expression Patterns in Canine Prostate and Urinary Bladder Tissue

The precise roles of ATRX and DAXX in human prostate carcinogenesis remain incompletely understood, and further research is needed to clarify their potential clinical relevance. Our results indicated that DAXX and ATRX undergo coordinated expression changes during malignant transformation, consistent with their known role as functional partners in chromatin regulation and telomere maintenance in human cancers [8]. In our cohort, DAXX and ATRX expression exhibited distinct patterns in prostate and urinary bladder cancers. This variation was not wholly unexpected, as reduced levels in human pancreatic neuroendocrine tumours [13,51] as well as increased expression in human high-grade meningiomas [11] have been described. Thus, the role of DAXX and ATRX in tumour biology must always be interpreted within the specific tumour type, both in human and veterinary oncology.

In our study, prostate carcinomas showed elevated nuclear expression of DAXX compared to non-malignant tissues, while ATRX showed no significant differences between the two groups. Furthermore, no significant differences between adenocarcinomas and prostatic urothelial carcinomas were observed. Our observation of DAXX overexpression in prostate cancer aligns with reports from human oncology, where strong DAXX expression has been associated with higher Gleason scores and increased tumour cell proliferation [22].

The ATRX expression in canine prostate carcinomas differs from human prostate cancer studies: While studies assessing ATRX nuclear protein expression in human prostate carcinomas are scarce, Coutinho-Camillo et al. reported significant ATRX mRNA downregulation in most cases [23]. A loss of ATRX expression could likewise promote genomic instability and thus facilitate tumour initiation [8]. However, ATRX overexpression may contribute to tumour progression through mechanisms such as autophagy inhibition, as proposed by Puto et al. for primary prostatic malignancies [52]. In conclusion, the role of ATRX in prostate tissue warrants further investigation in both humans and dogs.

In the urinary bladder, the expression levels of DAXX and ATRX did not differ significantly between non-malignant samples and UC. Interestingly, expression levels for both markers could differentiate Grade 2 from Grade 3 UC, with the more malignant Grade 3 UC showing a loss of expression. The reduced DAXX and ATRX expression in more malignant cases was consistent with findings in human oncology, particularly the study from Segersten et al., who reported that a loss of DAXX expression was significantly associated with a higher tumour stage (T2–4 compared to Ta and T1) in human urothelial bladder carcinomas [24].

### 4.3. Clinical Relevance in Human Medicine and Implications for Veterinary Medicine

In human medicine, DAXX and ATRX have gained increasing clinical relevance as molecular biomarkers. Diagnostically, assessment of their nuclear expression is used routinely to distinguish well-differentiated neuroendocrine tumours (NETs) and poorly differentiated neuroendocrine carcinomas (NECs). A loss of DAXX or ATRX expression strongly supports the diagnosis of NET, whereas retained nuclear expression is more typical for NEC [14]. For veterinary oncology, our findings indicate a potential diagnostic applicability in canine prostate cancer, as increased expression of DAXX could help distinguish carcinomas from non-malignant prostate tissues such as benign prostatic hyperplasia. This may be useful in challenging cases where conventional histopathological assessment is limited, such as when only small biopsies or fine-needle aspiration (FNA) material is available, or when artefacts impede accurate interpretation.

The prognostic value of both markers varies considerably depending on the type of tumour. A loss of expression for DAXX and ATRX in human medicine is associated with a worse prognosis in PanNENs [13,53] and urothelial bladder carcinomas [24]. A loss of ATRX may contribute to a better prognosis in patients with nasopharyngeal carcinoma [54]. Furthermore, high-grade serous ovarian carcinomas show poorer overall survival time when exhibiting elevated nuclear DAXX expression [55]. Our findings suggest a potential prognostic value of DAXX and ATRX in canine UC, which needs to be confirmed by clinically based follow-up studies with survival data. Although expression levels did not distinguish between non-malignant and malignant bladder tissues, UC showed a reduction in DAXX and ATRX expression with increasing tumour grade. Thus, both markers may help to identify biologically more aggressive UC and could serve as indicators of malignant progression.

Experimental therapeutic strategies targeting the inhibition of DAXX and ATRX overexpression are currently being explored in human medicine, although no clinically established inhibitors are available to date [56,57]. In contrast, the mutation or loss of ATRX expression may increase sensitivity to poly(ADP-ribose)-polymerase (PARP) inhibitors [58]. Notably, the PARP inhibitor Olaparib has already shown promising effects in canine cancer cell lines and may serve as a potential oral therapy in dogs [59]. Our findings suggest that targeting tumours with loss of expression for DAXX and/or ATRX may be of particular interest for treating highly aggressive UC in veterinary oncology. However, without correlating expression with genetic or functional data, it remains unclear whether the identified expression changes are causally linked to tumour biology or prognosis. Thus, this study can only offer fundamental insights into promising aspects for further studies to characterise these canine tumours in more detail.

A loss of DAXX and/or ATRX expression has been associated with activation of alternative lengthening of telomeres (ALT), a mechanism that enables cancer cells to maintain telomere length and sustain proliferation [60]. Therapeutic approaches targeting ALT-positive tumours, such as ATR-kinase inhibitors (e.g., Ceralasertib), are currently under investigation in human oncology [61,62]. Immunohistochemically detected loss of DAXX and/or ATRX serves as a practical surrogate marker for ALT in man. Kreilmeier et al. [18] detected ALT in 9.4% of analysed canine sarcomas. Interestingly, all ALT-positive sarcomas demonstrated a loss of ATRX expression and belonged to non-soft tissue entities. Similarly, Bicanova et al. reported ALT activity in 20% (10 of 50) of canine appendicular osteosarcoma [63]. Comparable studies on canine epithelial tumours are currently unavailable. Future studies should investigate whether UC with reduced DAXX and ATRX expression also shows an activation of the ALT pathway.

Personalised tumour therapy, precisely adapted to the individual patient’s and tumour’s genetic profile, is already well established in human medicine [64]. In contrast, veterinary oncology is still in the early stages of applying such approaches [65]. Therefore, it is crucial to deepen our understanding of the underlying mechanisms driving tumour development and progression and to further explore potential therapeutic options in veterinary medicine.

### 4.4. Conclusions

In conclusion, our pilot study demonstrated the expression of epithelial DAXX and ATRX in canine prostate and bladder tissues. The expression intensity of both markers was positively correlated and displayed organ-specific. Prostate carcinomas showed increased expression of DAXX compared with non-malignant tissues. In contrast, in the urinary bladder, DAXX and ATRX expression declined with increasing tumour grade (between grades 2 and 3). These findings provide novel insights into the potential role of DAXX and ATRX as biomarkers for these cancer types, laying the groundwork for future studies to explore their clinical significance in dogs. AI-assisted digital IHC evaluation enables a fully quantitative, automated, and reproducible analysis, which is particularly important for validating new biomarkers, such as DAXX and ATRX.

## Data Availability

The original contributions presented in the study are included in the article/Supplementary Materials, further inquiries can be directed to the corresponding author.

## Supplementary Materials

The following supporting information can be downloaded at: https://www.mdpi.com/article/10.3390/vetsci12121209/s1, Table S1: Overview of included cases examined for DAXX and ATRX expression.
