# Supplementary material for: DAXX and ATRX Expression in Canine Prostate and Bladder Cancer Identified by Immunohistochemistry—A Digital Quantitative Pilot Study
Source: Vet Sci. 2025 Dec 17;12(12):1209. doi: 10.3390/vetsci12121209 (PMC12737483; doi:10.3390/vetsci12121209)
Supplement: Supplementary file 1 [file vetsci-12-01209-s001.zip › vetsci-4003497-supplementary.pdf]

Supplementary Table S1. Overview of included cases examined for DAXX and ATRX expression.

CN	Sex	Breed	Age (years)	histological type; for carcinomas: predominant growth pattern, ITC if present	histological grade(1)	DAXX(2)	ATRX(3)
**prostatic carcinoma (18)**							
a) adenocarcinoma (12)					2-10		
1	m	Dachshund	11	mixed solid and tubular, ITC	9	173	58
2	mn	Bulldog, French	11	mixed solid and tubular, ITC	9	172	23
3	mn	Terrier, Jack Russell	nd	mixed solid and tubular	9	nd	nd
4	mn	Shi Tzu	10	mixed solid and tubular	9	191	nd
5	mn	Poodle	7	mixed solid and tubular, ITC	10	187	49
6	mn	Crossbreed	12	mixed solid and tubular	10	189	38
7	mn	Welsh Corgi	11	mixed solid and tubular	10	183	60
8	mn	Labrador Retriever	10	mixed solid and tubular	10	119	37
9	mn	Belgian Shepherd	9	tubular	10	163	20
10	m	Boxer	10	mixed solid and tubular	10	175	19
11	m	Dachshund	11	mixed solid and tubular	10	182	61
12	mn	Crossbreed	13	mixed solid and tubular	10	175	28
b) prostatic urothelial carcinoma (6)							
13	mn	Terrier, Yorkshire	7	urothelial		177	40
14	mn	Terrier, Jack Russell	nd	urothelial		192	66
15	mn	Poodle	12	urothelial		202	90
16	mn	Bullterrier, Staffordshire	10	urothelial		164	33
17	mn	Terrier, Jack Russell	12	urothelial		nd	nd
18	mn	Border Collie	12	urothelial		nd	nd
**non-malignant prostatic samples (10)**							
19	m	Terrier, West Highland White	10	healthy prostatic epithelium		166	18
20	m	Greyhound	3	healthy prostatic epithelium		117	11
21	m	Dogue de Bordeaux	4	healthy prostatic epithelium		132	16
22	m	Labrador Retriever	0	immature		136	22
23	m	Crossbreed	0	immature		123	24
24	m	Leonberger	0	immature		152	13
25	m	Cavalier King Charles Spaniel	0	immature		104	24
26	m	Bullterrier, Staffordshire	9	benign prostatic hyperplasia		178	54
27	m	Münsterländer, Small	8	benign prostatic hyperplasia		166	46
28	mn	Terrier, Border	13	atrophy		140	46
**urothelial carcinoma of the bladder (22)**					1-3		
29	mn	Beagle	11	solid	2	171	94
30	f	Terrier, Yorkshire	8	papillary	2	182	68
31	fn	Beagle	12	papillary	2	181	87
32	f	Beagle	10	papillary, ITC	2	175	53
33	f	Crossbreed	14	solid	2	152	84
34	fn	Cocker Spaniel	12	solid	2	190	97
35	f	Border Collie	11	papillary	2	185	41
36	fn	Terrier, Welsh	12	papillary	2	180	86
37	m	Terrier, Yorkshire	8	papillary	2	184	85
38	fn	Terrier, Scottish	7	mixed solid and papillary, ITC	2	184	89
39	mn	Terrier, Jack Russell	13	papillary	2	176	108
40	fn	Border Collie	10	solid	2	174	94
41	f	Sheepdog, Shetland	10	papillary	2	170	89
42	f	Crossbreed	12	solid	2	167	68
43	fn	Samojede	12	papillary	2	173	127
44	fn	Border Collie	11	papillary	2	168	41
45	m	Pointer	11	papillary	2	192	61
46	m	Bullterrier, Staffordshire	9	solid	3	170	78

47	fn	Nova S. Duck Tolling Retriever	11	papillary, ITC	3	119	38
48	fn	Terrier, Scottish	11	solid	3	139	64
49	m	Beagle	7	solid	3	127	22
50	mn	Terrier, West Highland White	9	papillary	3	140	16
**non-malignant bladder samples (6)**							
51	f	Petit Brabançon	9	healthy urothel		158	50
52	m	Terrier, Welsh	10	healthy urothel		167	35
53	fn	Bullterrier, Staffordshire	nd	hyperplasia		158	59
54	m	Miniature Schnauzer	13	papillom		176	98
55	m	Irish Setter	4	papillom		175	82
56	m	Terrier, Yorkshire	8	papillom		nd	23

Histological grade(1): UC Grade 1-3 based on Vallie et al. (Valli et al. 1995); PC Score 2-10 based on Palmieri et al. (Palmieri und Grieco 2015).

DAXX(2) and ATRX(3): expression levels; aggregated and inverted mean HDAB-DAB values from all nuclei epithelium as generated by analysis with Visiopharm software.

Abbreviations: CN, case number; f, female; fn, female neutered; m, male; mn, male neutered; ITC, intravascular tumour cell detection; nd, no data available. Age = age at the time of sampling.
